# The Role of NF-κB Triggered Inflammation in Cerebral Ischemia

**DOI:** 10.3389/fncel.2021.633610

**Published:** 2021-05-10

**Authors:** Teresa Jover-Mengual, Jee-Yeon Hwang, Hyae-Ran Byun, Brenda L. Court-Vazquez, José M. Centeno, María C. Burguete, R. Suzanne Zukin

**Affiliations:** ^1^Dominick P. Purpura Department of Neuroscience, Albert Einstein College of Medicine, New York, NY, United States; ^2^Unidad Mixta de Investigación Cerebrovascular, Instituto de Investigación Sanitaria La Fe-Universidad de Valencia, Valencia, Spain; ^3^Departamento de Fisiología, Universidad de Valencia, Valencia, Spain; ^4^Department of Pharmacology, Creighton University School of Medicine, Omaha, NE, United States

**Keywords:** NF-κB, inflammation, cerebral ischemia, neuroprotection, neurodegeneration

## Abstract

Cerebral ischemia is a devastating disease that affects many people worldwide every year. The neurodegenerative damage as a consequence of oxygen and energy deprivation, to date, has no known effective treatment. The ischemic insult is followed by an inflammatory response that involves a complex interaction between inflammatory cells and molecules which play a role in the progression towards cell death. However, there is presently a matter of controversy over whether inflammation could either be involved in brain damage or be a necessary part of brain repair. The inflammatory response is triggered by inflammasomes, key multiprotein complexes that promote secretion of pro-inflammatory cytokines. An early event in post-ischemic brain tissue is the release of certain molecules and reactive oxygen species (ROS) from injured neurons which induce the expression of the nuclear factor-kappaB (NF-κB), a transcription factor involved in the activation of the inflammasome. There are conflicting observations related to the role of NF-κB. While some observe that NF-κB plays a damaging role, others suggest it to be neuroprotective in the context of cerebral ischemia, indicating the need for additional investigation. Here we discuss the dual role of the major inflammatory signaling pathways and provide a review of the latest research aiming to clarify the relationship between NF-κB mediated inflammation and neuronal death in cerebral ischemia.

## Introduction

Cerebral ischemia, due to insufficient blood supply to the brain, leads to neurodegeneration as a consequence of deprivation of oxygen and energy needed for the metabolic requirements of the brain. Ischemia can occur in two forms: focal ischemia (stroke), affecting a specific area of the brain when a particular cerebral artery is occluded, or global ischemia, affecting the entire brain and typically caused when cardiac activity comes to a halt, as in a cardiac arrest or during surgery (Zhang et al., 2016).

This devastating disease is one of the leading causes of death as well as a major cause of adult disability from neurological dysfunction (Katan and Luft, 2018; World Health Organization, 2018).

Lack of oxygen and glucose supply after cerebral ischemia leads to neurons and glial cells being unable to maintain their ionic homeostasis (Murphy, 2015). This triggers a complex series of events known as the ischemic cascade, for review see Iadecola and Anrather (2011) and Azad et al. (2016). Briefly, the disruption of the cellular ionic gradients results in an excess release of glutamate, causing an increase in the intracellular calcium influx and excitotoxicity that initiates necrotic and apoptotic cell death pathways. During these cell death processes, reactive oxygen species (ROS) can be generated by cellular ionic imbalances, which mediates membrane, mitochondrial, and DNA damage resulting in protein misfolding and inflammation (Iadecola and Anrather, 2011). Importantly, reperfusion after cerebral ischemia also increases the production of ROS and the inflammatory response, worsening neuronal injury (Wu et al., 2018). All these processes seriously damage neurons, glia and endothelial cells.

Despite major recent advances in the understanding of the pathogenesis of cerebral ischemia, to date, there is no known effective treatment. A large number of neuroprotective strategies have shown disappointing results in clinical trials due to lack of efficacy or undesirable side effects (Ginsberg, 2008). At present, the most effective approach to reduce damage from cerebral ischemia is the early restoration of cerebral blood flow (Hankey, 2017). However, rapid revascularization after cerebral ischemia can evoke secondary injury caused by the inflammatory response. This cascade of events involves a complex interaction between inflammatory cells and other molecules in the brain, resulting in the release of inflammatory mediators which play a role in the progression of cell damage and death (Shichita et al., 2014; Khoshnam et al., 2017). The production of multiple stimuli from injured ischemic neurons such as the influx of calcium, the formation of ROS, and various other molecules are early events in the postischemic brain that induce the expression, among others, of the nuclear factor-kappaB (NF-κB), a proinflammatory transcription factor involved in the activation of the inflammasome (Shih et al., 2015) and a central player in triggering the inflammatory response (Savage et al., 2012; Baroja-Mazo et al., 2014; Gao et al., 2017). This review article summarizes the major inflammatory signaling pathway and the role of NF-κB in cerebral ischemia-induced-neuronal death.

## The Inflammatory Response in Cerebral Ischemia

### How Does Inflammation Occur After Cerebral Ischemia?

Inflammation is a crucial response of the innate immune system that is activated by peripheral circulatory cells (Benakis et al., 2014) and cerebral ischemia-induced neuronal damage to restore homeostasis, tissue repair, and reorganization (Anrather and Iadecola, 2016). However, chronic inflammatory response leads to secondary injury after cerebral ischemia, which is why these processes are so difficult to understand (Kawabori and Yenari, 2015; Banjara and Ghosh, 2017).

**Figure 1:**
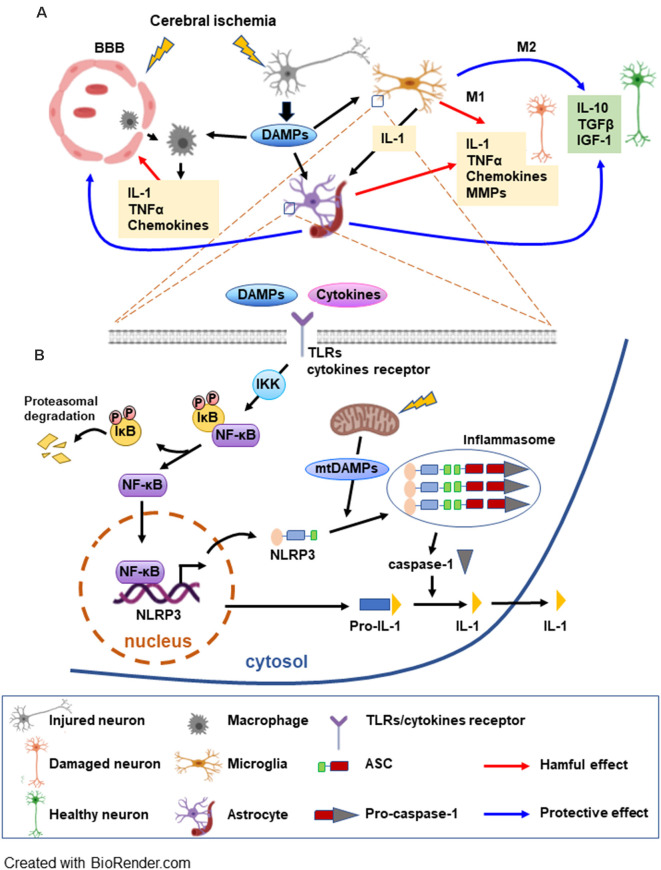
Schematic overview of the postischemic inflammatory response and NF-κB signaling pathways mediating NLRP3 inflammasome activation. **(A)** Cerebral ischemia triggers the disruption of the blood brain barrier (BBB) allowing for the macrophages to migrate to the ischemic area. Injured neurons release DAMPs soon after the onset of ischemia, which can be detected by microglia, astrocytes, and macrophages initiating an inflammatory response. The inflammatory molecules released by the glia have deleterious as well beneficial effects on neurons. M1 microglia generate interleukin (IL)-1β and tumor necrosis factor (TNF) which further induce cytokine and chemokine release from astrocytes. M1 microglia also release MMPs, enhancing BBB disruption and worsening brain damage. A beneficial role of glia consists of producing anti-inflammatory cytokines and trophic molecules. Additionally, astrocytes can be involved in the repair of the BBB. **(B)** DAMPs and cytokines trigger TLR/cytokine receptor signaling, leading to NF-κB activation. NF-κB, a major factor in the inflammasome priming phase, is activated upon phosphorylation of the inhibitor IκB by IKK. NF-κB can then translocate to the nucleus to induce transcription of the inflammasome complex elements. DAMPs released by mitochondria (mtDAMPs) constitute the signal that triggers the assembly and activation of the inflammasome. Caspase-1 is activated by the inflammasome and can transform the inactive pro-IL-1 into active IL-1 which can be released (see text for abbreviations).

After cerebral artery occlusion, ROS production activates endothelial cells with expression of adhesion molecules and disruption of the blood-brain barrier (BBB; Abdullahi et al., 2018; Bayraktutan, 2019) which allows infiltration of leukocytes (Yang et al., 2019). Infiltrated leukocytes and microglia are accumulated in the ischemic brain tissue releasing inflammatory mediators. Infiltrated leukocytes such as neutrophils and T lymphocytes produce pro-inflammatory cytokines and cytotoxic substances, damaging cerebral tissue following ischemia (Garcia-Bonilla et al., 2014; Strecker et al., 2017). The activated microglia, the brain’s resident macrophages, induce change in cell morphology to either pro-inflammatory M1 or anti-inflammatory M2 phenotype (Lee et al., 2014). In the ischemic brain, M1 microglia initiates post-ischemic inflammation by generating interleukin (IL)-1β, ROS and tumor necrosis factor (TNF) which further induces cytokine and chemokine release from astrocytes and endothelial cells (Anrather and Iadecola, 2016; Ma et al., 2017) which in turn, exacerbate brain damage. In addition, M1 microglia also releases matrix metalloproteinases (MMPs) which further enhance BBB disruption (Shekhar et al., 2018). In contrast, M2 microglia releases anti-inflammatory cytokines and growth factors which mitigate ischemia-induced brain damage (Xiong et al., 2016; Kanazawa et al., 2017; Figure 1A).

As an early postischemic event, the damage-associated molecular patterns (DAMPs) are released. DAMPs are the products of injured or stressed neurons and glial cells that are originally intended to activate repair mechanisms (Patel, 2018). DAMPs include diverse types of molecules, such as endogenous proteins, nucleic acids, and metabolites that trigger an immune response leading to cell damage (Gadani et al., 2015). Endogenous protein DAMPs, high mobility group protein B1 (HMGB1; Deng et al., 2019), IL-33, IL-1α, Ca^2 +^ -binding S100 proteins (Bertheloot and Latz, 2017), heat shock proteins (HSPs; Tamura et al., 2012), and peroxiredoxin (PRXs; Nakamura and Shichita, 2019) are released after cell damage and initiate inflammation. Another group of DAMPs includes mitochondrial DNA (mtDNA; West and Shadel, 2017), nucleotide derivatives such as ATP, uric acid, and ROS (Heil and Land, 2014; Patel, 2018). DAMPs and cytokines trigger activation of toll-like receptors (TLRs) which are one of the several transmembrane pattern-recognition receptors and play a pivotal role in the inflammatory response after brain injury (Kumar, 2019). Activated TLRs increase NLRP3 [nucleotide-binding oligomerization domain (NOD)-like receptor protein 3] expression through the NF-κB signaling pathway. Together with the adaptor ASC [apoptosis-associated speck-like protein containing CARD (caspase recruitment domain)] and procaspase-1, NLRP3 forms a caspase-1 activating complex known as the NLRP3 inflammasome (Gao et al., 2017), then the activated caspase-1 converts the pro-form to mature form of pro-inflammatory cytokine IL-1 and IL-18 (Figure 1B). We discuss the inflammation complex in more depth in the “Proinflammatory Role of Inflammasome” section.

### Proinflammatory Role of Inflammasome

Inflammasome is a multiprotein complex that mediates the secretion of inflammatory cytokines by the immune system and nerve cells, after perception of danger signals, triggering cell death in cerebral ischemia (Gao et al., 2017; Mohamadi et al., 2018). Among the different receptor proteins that can form inflammasomes, NLRP3 is one of the best characterized component of an NLR family (Wen et al., 2013; Broz and Dixit, 2016). NLRP3 inflammasome is formed by NACHT, a nucleotide binding and oligomerization domain, a C-terminal leucine-rich repeat (LRR), N-terminal adaptor protein ASC [that contains a pyrin domain (PYD)] and procaspase-1 (Schroder and Tschopp, 2010; Voet et al., 2019; Feng et al., 2020).

Inflammasome complex activation and production of cytokines are done by a well-regulated mechanism that requires two phases. The priming phase involves activation of the transmembrane TLRs by DAMPs from damaged and dead cells, resulting in the upregulation of the NF-κB and MAPK signaling pathways followed by the transcription of the inflammasome protein and pro-IL-1β and pro-IL-18 (Bauernfeind et al., 2011; Fann et al., 2013). The second phase is the activation of the inflammasome complex by DAMPs, such mitochondrial ROS, triggering oligomerization of the NLRP3 receptors and recruitment of ASC (Rajanbabu et al., 2015). The ASC binds to procaspase-1 resulting in its cleavage and conversion to active caspase-1 that in turn triggers the activation of IL-1 and IL-18 initiating inflammation (Schroder and Tschopp, 2010; Sutterwala et al., 2014; Broz and Dixit, 2016; Gong et al., 2018).

NLRP3 inflammasome is expressed in microglia and neurons (Song et al., 2017; Fann et al., 2018). The astrocytes secrete pro-inflammatory cytokines, however, it has been reported that NLRP3 inflammasome is not functional in mouse astrocytes (Gustin et al., 2015). In contrast, Freeman et al. (2017) provided direct evidence for the function of NLRP3 in astrocytes. Neuronal and glial cell death after cerebral ischemia is correlated with increased inflammasome activity (Fann et al., 2013; Ma et al., 2019). Moreover, cerebral ischemia-induced neuronal cell death and inflammasome expression are attenuated by NF-κB and MAPK inhibitors (Fann et al., 2018; Figure 1B).

### Anti-inflammatory Response by Glial Cells: a Beneficial Effect of Inflammatory Process

Cerebral ischemia induces cell death that activates the inflammatory response, secretes pro-inflammatory cytokines, and promotes further brain damage *via* activation of glial cells (Iadecola and Anrather, 2011; Kawabori and Yenari, 2015; Banjara and Ghosh, 2017). However, depending on the context and timing, activated glial cells also play a beneficial role by removing dead cells and promoting recovery (Jayaraj et al., 2019; Sakai and Shichita, 2019). Resolution of inflammation and tissue repair is a contribution of activated M2 microglia which become phagocytic and clear tissue damage (Iadecola and Anrather, 2011; Zhang, 2019). They also release anti-inflammatory cytokines such as TGF-β, IL-4, IL-10, IL-13, growth factors, progranulin which represses inflammation and alleviates brain damage after ischemic insults (Xiong et al., 2016; Kanazawa et al., 2017) and produce insulin-like growth factor (IGF)-1 during the reparative phase of the inflammation (Amantea et al., 2014). Moreover, brain injury after cerebral ischemia is exacerbated by the deletion of proliferating microglia (Lalancette-Hébert et al., 2007) and is attenuated by administration of exogenous microglia (Imai et al., 2007).

Astroglia also play a beneficial role in the process of inflammation. Several studies suggest that the astrocytes reactions are involved in the repair and neuroprotection by reconstructing the BBB (del Zoppo, 2009) and delivering neurotrophic factors (Shen et al., 2010) in cerebral ischemia. Ischemia activates astrocytes and increases glial fibrillary acidic protein (GFAP) expression, reactive gliosis, and glial scar with both detrimental and beneficial functions (Liu and Chopp, 2016; Pekny et al., 2019). Astrocytes form functional borders that regulate the inflammatory cells, restraining the extent of neurotoxic inflammation (Sofroniew, 2015). The deletion of reactive astrocytes has been shown to frustrate the BBB repair, increasing inflammation and tissue damage after cerebral ischemia (Li et al., 2008). On the other hand, induction of astrocyte proliferation is correlated with neuroprotection in cerebral ischemia models (Keiner et al., 2008). These studies indicate that inflammatory cells in the CNS have a dual role, both beneficial and deleterious by activating and repressing inflammation in a time- and context-dependent manner (Figure 1A).

## NF-κB in The Brain

The pleiotropic transcription factor NF-κB, as a key regulator of numerous cellular signaling pathways, is involved in cell survival, immune responses, and inflammation (Shih et al., 2015). NF-κB contains dimers of the Rel protein family that includes RelA (p65), p50, p52, Rel B, and C-Rel. It is amply expressed in neurons and glial cells as a heterodimer of RelA (p65) and p50 subunits (Ridder and Schwaninger, 2009). Under resting conditions, NF-κB dimers are retained in the cytoplasm, in an inactive state, by interacting with its endogenous inhibitor IκB to prevent nuclear translocation (Napetschnig and Wu, 2013).

Two major signaling pathways can activate NF-κB, canonical and noncanonical pathways. The most researched canonical NF-κB pathway is activated by many different exogenous and endogenous factors, including DAMPs, cytokines, ROS, growth factors, immune-related receptors, and other signals such as neurotransmitters and nerve growth factors. These factors are perceived by specific membrane receptors such as TLRs and IL-1 receptors and many others that trigger the activation of the IKK (IκB upstream kinase). IKK activation induces phosphorylation, ubiquitination, and degradation of the IκB resulting in NF-κB translocation to the nucleus and stimulation of the transcription of many different genes (Ridder and Schwaninger, 2009). The noncanonical NF-κB pathway responds to ligands of members of the TNF family receptor and does not rely on IκB degradation but rather depends on the processing of the NF-κB precursor (Sun, 2012). Canonical NF-κB pathway is involved in both acute and chronic inflammatory responses and in cell survival whereas noncanonical NF-κB pathway cooperates with canonical NF-κB pathway in regulating different immune functions (Sun, 2017).

NF-κB is highly expressed in all cell types in the brain with different functions. NF-κB is observed in neuronal processes and in isolated synapses (Meffert et al., 2003). It is constitutively activated in hippocampal neurons (Fridmacher et al., 2003), in GABAergic interneurons (O’Mahony et al., 2006) as well as in other types of neurons from various regions of the brain (Shih et al., 2015). Many different stimuli have been shown to activate neuronal NF-κB including inflammatory mediators, neuronal growth factor (NGF), brain-derived growth factor (BDNF), and excitatory neurotransmitters (Snow and Albensi, 2016). Neuronal NF-κB is activated through NMDA glutamate receptors and L-type voltage calcium channels in neurons from the hippocampus, cerebellum, and the cortex (Meffert and Baltimore, 2005). Under physiological conditions, NF-κB promotes synaptic activity which maintains the ideal basal constitutive level in neurons and is repressed by IκB in an autoregulatory manner (Dresselhaus and Meffert, 2019). Trophic factor deprivation decreases NF-κB activity in cerebellar neurons by increasing the NF-κB inhibitor IκB (Kovács et al., 2004).

NF-κB signaling disruption increases neurodegeneration in excitatory neurons (Fridmacher et al., 2003) and in GABAergic interneurons indicating a role for NF-κB in synaptic plasticity and memory (O’Mahony et al., 2006). On the other hand, NF-κB activation promotes dendritic spines while the inhibition of NF-κB activity reduces dendritic spines suggesting a role for NF-κB in the excitatory synaptic function (Boersma et al., 2011). NF-κB regulates a large number of gene targets implicated in neuronal synaptic plasticity that are involved in behavior (Dresselhaus and Meffert, 2019). Additionally, several studies cite evidence that the activation of NF-κB is required for neuronal survival (Bhakar et al., 2002; Kaltschmidt et al., 2005; Mattson and Meffert, 2006).

In contrast, NF-κB has low basal activity in glial cells and there is no report indicating that glial NF-κB can be activated under resting state or by neurotransmitters as in the case with neurons. However, it has been reported that microglial NF-κB appears to have a role in the development and regulation of neuronal excitability and synaptic plasticity (Kyrargyri et al., 2015). Astrocytes have an important function in forming the BBB as well as in neuronal support and repair. Moreover, astrocytes are involved in the termination of excitatory signals by removing glutamate from synapses that rely on NF-κB activation (Ghosh et al., 2011).

## Role of NF-κB in Cerebral Ischemia

The main controversy is whether NF-κB activation has a neuroprotective role or its function as an inflammatory mediator exacerbates neuronal damage after cerebral ischemia. NF-κB regulates caspase inhibitors, TNF-receptor associate factor (TRAF), and the Bcl-2 family proteins (Wang et al., 1998, 1999) increasing cell survival. These antiapoptotic and survival-promoting genes can code for resistance to cell death under pathological conditions (Yang et al., 2007; Mettang et al., 2018). Overexpression of p65 rescued apoptotic cortical neurons while selective inhibition of NF-κB enhanced damage (Bhakar et al., 2002) suggests that NF-κB is involved in an anti-apoptotic function in neurons. In contrast, other studies propose that the neuronal activation of NF-κB contributes to the death of neurons after cerebral ischemia (Nurmi et al., 2004; Zhang et al., 2005). NF-κB is activated in degenerating hippocampal CA1 neurons 24–72 h after global ischemia (Clemens et al., 1997) and 24 h after focal ischemia in association with reactive glial cells, and at later times, in rats (Gabriel et al., 1999). Moreover, infarct volumes are decreased in experiments with mice lacking the p50 subunit of NF-κB indicating that NF-κB activation could promote neuronal death after ischemia (Nurmi et al., 2004). NF-κB has different functions that may depend on the NF-κB complex composition (Lanzillotta et al., 2015).

Besides neurons, NF-κB signaling pathways in glia can be activated in response to injury through TLR signaling, increasing the production of inflammatory mediators (Dresselhaus and Meffert, 2019). Microglia, the immune resident macrophages in the brain, can become activated by an NF-κB-mediated mechanism under injury conditions. This activation can trigger a release of large amounts of pro-inflammatory cytokines, such as TNFα, IL-1β, and ROS (Block et al., 2007) that can generate secondary neurotoxicity aggravating neurodegeneration. Astrocyte activation by TLR is involved in innate immune reactions secreting IL-1β and IL-6 (Kopitar-Jerala, 2015) and after cerebral ischemia, astrocytes are involved in inflammation mediated by NF-κB (Deng et al., 2018). Astroglial NF-κB inhibition induces a decrease in the expression of chemokines and leukocyte infiltration showing a role in the intracellular pro-inflammatory activation (Gonzalez-Reyes and Rubiano, 2018).

Neurotoxicity mediated by glial cells may give an explanation of the neuronal damage and NF-κB activity decreases after cerebral ischemia (Mattson and Meffert, 2006). Considerable evidence now suggests that neuronal NF-κB has an anti-apoptotic and survival role while its activation in glial cells is involved in inflammation and cell death. NF-κB’s possible protective role in neurons should be separated from the most likely probable degenerative role in glia (Kaltschmidt et al., 2005; Dresselhaus and Meffert, 2019).

## Conclusions

Inflammation is a central component in the progression of cell damage after cerebral ischemia that involves neurons, glia, and immune cells. Growing evidence indicates that inflammation has a dual role: while the acute inflammatory response seems to aggravate an ischemic injury, the recovery and tissue repair depend on later inflammatory processes. Moreover, it is accepted that pro-inflammatory glial and immune cells in the acute phase of ischemia may differentiate into anti-inflammatory cells that contribute to the regeneration processes during the chronic phase of cerebral ischemia. It is well established that NF-κB has a crucial role in cell survival and inflammation. All brain cell types express NF-κB, but under basal conditions it is constitutively activated in neurons, while it has little activity in glial cells, showing different functions in different nerve cells. Neuronal NF-κB has a potential preservative role that should be differentiated from the degenerative role it plays in glial cells under ischemic conditions. Based on all the above, an ideal and successful anti-inflammatory treatment should attack and inhibit, in a very selective manner, the deleterious constituents of the inflammatory process during cerebral ischemia and, simultaneously, enhance the beneficial aspects of the inflammatory response. Additionally, the treatment should selectively target, in its actions, different cell types. In the context of cerebral ischemia, a deeper understanding of the step-by-step progression of the inflammatory cascade as well as the specific functions of NF-κB, in the different cell types, is crucial for the development of an effective therapeutic regimen.

## Author Contributions

TJ-M, J-YH and MCB contributed to the design and draft of the manuscript. H-RB and JMC conducted the literature review. BC-V contributed to the preparation of the figure. RSZ critically revised and edited the manuscript content. All authors contributed to the article and approved the submitted version.

## Conflict of Interest

The authors declare that the research was conducted in the absence of any commercial or financial relationships that could be construed as a potential conflict of interest.

## References

[B1] Abdullahi W. Tripathi D. Ronaldson P. T. (2018). Blood-brain barrier dysfunction in ischemic stroke: targeting tight junctions and transporters for vascular protection. Am. J. Physiol. Cell Physiol. 315, C343–C356. 10.1152/ajpcell.00095.2018 29949404 PMC6171039

[B2] Amantea D. Tassorelli C. Petrelli F. Certo M. Bezzi P. Micieli G.. (2014). Understanding the multifaceted role of inflammatory mediators in ischemic stroke. Curr. Med. Chem. 21, 2098–2117. 10.2174/0929867321666131227162634 24372219

[B3] Anrather J. Iadecola C. (2016). Inflammation and stroke: an overview. Neurotherapeutics 13, 661–670. 10.1007/s13311-016-0483-x 27730544 PMC5081118

[B4] Azad T. D. Veeravagu A. Steinberg G. K. (2016). Neurorestoration after stroke. Neurosurg. Focus 40:E2. 10.3171/2016.2.FOCUS15637 27132523 PMC4916840

[B5] Banjara M. Ghosh C. (2017). Sterile neuroinflammation and strategies for therapeutic intervention. Int. J. Inflamm. 2017:8385961. 10.1155/2017/8385961 28127491 PMC5239986

[B6] Baroja-Mazo A. Martín-Sánchez F. Gomez A. I. Martínez C. M. Amores-Iniesta J. Compan V.. (2014). The NLRP3 inflammasome is released as a particulate danger signal that amplifies the inflammatory response. Nat. Immunol. 15, 738–748. 10.1038/ni.2919 24952504

[B7] Bauernfeind F. Ablasser A. Bartok E. Kim S. Schmid-Burgk J. Cavlar T.. (2011). Inflammasomes: current understanding and open questions. Cell. Mol. Life Sci. 68, 765–783. 10.1007/s00018-010-0567-4 21072676 PMC11114650

[B8] Bayraktutan U. (2019). Endothelial progenitor cells: potential novel therapeutics for ischaemic stroke. Pharmacol. Res. 144, 181–191. 10.1016/j.phrs.2019.04.017 31004788

[B9] Benakis C. Garcia-Bonilla L. Iadecola C. Anrather J. (2014). The role of microglia and myeloid immune cells in acute cerebral ischemia. Front. Cell. Neurosci. 8:461. 10.3389/fncel.2014.00461 25642168 PMC4294142

[B10] Bertheloot D. Latz E. (2017). HMGB1, IL-1α, IL-33 and S100 proteins: dual-function alarmins. Cell. Mol. Immunol. 14, 43–64. 10.1038/cmi.2016.34 27569562 PMC5214941

[B11] Bhakar A. L. Tannis L.-L. Zeindler C. Russo M. P. Jobin C. Park D. S.. (2002). Constitutive nuclear factor-κ B activity is required for central neuron survival. J. Neurosci. 22, 8466–8475. 10.1523/JNEUROSCI.22-19-08466.2002 12351721 PMC6757785

[B12] Block M. L. Zecca L. Hong J.-S. (2007). Microglia-mediated neurotoxicity: uncovering the molecular mechanisms. Nat. Rev. Neurosci. 8, 57–69. 10.1038/nrn2038 17180163

[B13] Boersma M. C. Dresselhaus E. C. De Biase L. M. Mihalas A. B. Bergles D. E. Meffert M. K. (2011). A requirement for nuclear factor-κB in developmental and plasticity-associated synaptogenesis. J. Neurosci. 31, 5414–5425. 10.1523/JNEUROSCI.2456-10.2011 21471377 PMC3113725

[B14] Broz P. Dixit V. M. (2016). Inflammasomes: mechanism of assembly, regulation and signalling. Nat. Rev. Immunol. 16, 407–420. 10.1038/nri.2016.58 27291964

[B15] Clemens J. A. Stephenson D. T. Dixon E. P. Smalstig E. B. Mincy R. E. Rash K. S.. (1997). Global cerebral ischemia activates nuclear factor-κB prior to evidence of DNA fragmentation. Mol. Brain Res. 48, 187–196. 10.1016/s0169-328x(97)00092-2 9332715

[B16] del Zoppo G. J. (2009). Inflammation and the neurovascular unit in the setting of focal cerebral ischemia. Neuroscience 158, 972–982. 10.1016/j.neuroscience.2008.08.028 18824084 PMC2665879

[B18] Deng Y.-L. Ma Y.-L. Zhang Z.-L. Zhang L.-X. Guo H. Qin P.. (2018). Astrocytic N-Myc downstream-regulated gene-2 is involved in nuclear transcription factor κB-mediated inflammation induced by global cerebral ischemia. Anesthesiology 128, 574–586. 10.1097/ALN.0000000000002044 29252510

[B17] Deng M. Scott M. J. Fan J. Billiar T. R. (2019). Location is the key to function: HMGB1 in sepsis and trauma-induced inflammation. J. Leukoc. Biol. 106, 161–169. 10.1002/JLB.3MIR1218-497R 30946496 PMC6597316

[B19] Dresselhaus E. C. Meffert M. K. (2019). Cellular specificity of NF-κB function in the nervous system. Front. Immunol. 10:1043. 10.3389/fimmu.2019.01043 31143184 PMC6520659

[B20] Fann D. Y.-W. Lee S.-Y. Manzanero S. Chunduri P. Sobey C. G. Arumugam T. V. (2013). Pathogenesis of acute stroke and the role of inflammasomes. Ageing Res. Rev. 12, 941–966. 10.1016/j.arr.2013.09.004 24103368

[B21] Fann D. Y.-W. Lim Y.-A. Cheng Y.-L. Lok K.-Z. Chunduri P. Baik S.-H.. (2018). Evidence that NF-κB and MAPK signaling promotes NLRP inflammasome activation in neurons following ischemic stroke. Mol. Neurobiol. 55, 1082–1096. 10.1007/s12035-017-0394-9 28092085

[B22] Feng Y.-S. Tan Z.-X. Wang M.-M. Xing Y. Dong F. Zhang F. (2020). Inhibition of NLRP3 inflammasome: a prospective target for the treatment of ischemic stroke. Front. Cell. Neurosci. 14:155. 10.3389/fncel.2020.00155 32581721 PMC7283578

[B23] Freeman L. Guo H. David C. N. Brickey W. J. Jha S. Ting J. P. (2017). NLR members NLRC4 and NLRP3 mediate sterile inflammasome activation in microglia and astrocytes. J. Exp. Med. 214, 1351–1370. 10.1084/jem.20150237 28404595 PMC5413320

[B24] Fridmacher V. Kaltschmidt B. Goudeau B. Ndiaye D. Rossi F. M. Pfeiffer J.. (2003). Forebrain-specific neuronal inhibition of nuclear factor-κB activity leads to loss of neuroprotection. J. Neurosci. 23, 9403–9408. 10.1523/JNEUROSCI.23-28-09403.2003 14561868 PMC6740573

[B25] Gabriel C. Justicia C. Camins A. Planas A. M. (1999). Activation of nuclear factor-κB in the rat brain after transient focal ischemia. Mol. Brain Res. 65, 61–69. 10.1016/s0169-328x(98)00330-1 10036308

[B26] Gadani S. P. Walsh J. T. Lukens J. R. Kipnis J. (2015). Dealing with danger in the CNS: the response of the immune system to injury. Neuron 87, 47–62. 10.1016/j.neuron.2015.05.019 26139369 PMC4491143

[B27] Gao L. Dong Q. Song Z. Shen F. Shi J. Li Y. (2017). NLRP3 inflammasome: a promising target in ischemic stroke. Inflamm. Res. 66, 17–24. 10.1007/s00011-016-0981-7 27576327

[B28] Garcia-Bonilla L. Moore J. M. Racchumi G. Zhou P. Butler J. M. Iadecola C.. (2014). Inducible nitric oxide synthase in neutrophils and endothelium contributes to ischemic brain injury in mice. J. Immunol. 193, 2531–2537. 10.4049/jimmunol.1400918 25038255 PMC4147670

[B29] Ghosh M. Yang Y. Rothstein J. D. Robinson M. B. (2011). Nuclear factor-κB contributes to neuron-dependent induction of glutamate transporter-1 expression in astrocytes. J. Neurosci. 31, 9159–9169. 10.1523/JNEUROSCI.0302-11.2011 21697367 PMC3138498

[B30] Ginsberg M. D. (2008). Neuroprotection for ischemic stroke: past, present and future. Neuropharmacology 55, 363–389. 10.1016/j.neuropharm.2007.12.007 18308347 PMC2631228

[B31] Gong Z. Pan J. Shen Q. Li M. Peng Y. (2018). Mitochondrial dysfunction induces NLRP3 inflammasome activation during cerebral ischemia/reperfusion injury. J. Neuroinflammation 15:242. 10.1186/s12974-018-1282-6 30153825 PMC6114292

[B32] Gonzalez-Reyes R. E. Rubiano M. G. (2018). Astrocyte’s RAGE: more than just a question of mood. Cent. Nerv. Syst. Agents Med. Chem. 18, 39–48. 10.2174/1871524916999160505105121 27149992

[B33] Gustin A. Kirchmeyer M. Koncina E. Felten P. Losciuto S. Heurtaux T.. (2015). NLRP3 inflammasome is expressed and functional in mouse brain microglia but not in astrocytes. PLoS One 10:e0130624. 10.1371/journal.pone.0130624 26091541 PMC4474809

[B34] Hankey G. J. (2017). Stroke. Lancet 389, 641–654. 10.1016/S0140-6736(16)30962-X 27637676

[B35] Heil M. Land W. G. (2014). Danger signals—damaged-self recognition across the tree of life. Front. Plant Sci. 5:578. 10.3389/fpls.2014.00578 25400647 PMC4215617

[B36] Iadecola C. Anrather J. (2011). The immunology of stroke: from mechanisms to translation. Nat. Med. 17, 796–808. 10.1038/nm.2399 21738161 PMC3137275

[B37] Imai F. Suzuki H. Oda J. Ninomiya T. Ono K. Sano H.. (2007). Neuroprotective effect of exogenous microglia in global brain ischemia. J. Cereb. Blood Flow Metab. 27, 488–500. 10.1038/sj.jcbfm.9600362 16820801

[B38] Jayaraj R. L. Azimullah S. Beiram R. Jalal F. Y. Rosenberg G. A. (2019). Neuroinflammation: friend and foe for ischemic stroke. J. Neuroinflammation 16:142. 10.1186/s12974-019-1516-2 31291966 PMC6617684

[B39] Kaltschmidt B. Widera D. Kaltschmidt C. (2005). Signaling *via* NF-κB in the nervous system. Biochim. Biophys. Acta 1745, 287–299. 10.1016/j.bbamcr.2005.05.009 15993497

[B40] Kanazawa M. Ninomiya I. Hatakeyama M. Takahashi T. Shimohata T. (2017). Microglia and monocytes/macrophages polarization reveal novel therapeutic mechanism against stroke. Int. J. Mol. Sci. 18:2135. 10.3390/ijms18102135 29027964 PMC5666817

[B41] Katan M. Luft A. (2018). Global burden of stroke. Semin. Neurol. 38, 208–211. 10.1055/s-0038-1649503 29791947

[B42] Kawabori M. Yenari M. A. (2015). Inflammatory responses in brain ischemia. Curr. Med. Chem. 22, 1258–1277. 10.2174/0929867322666150209154036 25666795 PMC5568039

[B43] Keiner S. Wurm F. Kunze A. Witte O. W. Redecker C. (2008). Rehabilitative therapies differentially alter proliferation and survival of glial cell populations in the perilesional zone of cortical infarcts. Glia 56, 516–527. 10.1002/glia.20632 18240310

[B44] Khoshnam S. E. Winlow W. Farzaneh M. Farbood Y. Moghaddam H. F. (2017). Pathogenic mechanisms following ischemic stroke. Neurol. Sci. 38, 1167–1186. 10.1007/s10072-017-2938-1 28417216

[B45] Kopitar-Jerala N. (2015). Innate immune response in brain, NF-κ B signaling and cystatins. Front. Mol. Neurosci. 8:73. 10.3389/fnmol.2015.00073 26696821 PMC4673337

[B46] Kovács A. D. Chakraborty-Sett S. Ramirez S. H. Sniderhan L. F. Williamson A. L. Maggirwar S. B. (2004). Mechanism of NF-κB inactivation induced by survival signal withdrawal in cerebellar granule neurons. Eur. J. Neurosci. 20, 345–352. 10.1111/j.1460-9568.2004.03493.x 15233744

[B47] Kumar V. (2019). Toll-like receptors in the pathogenesis of neuroinflammation. J. Neuroimmunol. 332, 16–30. 10.1016/j.jneuroim.2019.03.012 30928868

[B48] Kyrargyri V. Vega-Flores G. Gruart A. Delgado-García J. M. Probert L. (2015). Differential contributions of microglial and neuronal IKKβ to synaptic plasticity and associative learning in alert behaving mice. Glia 63, 549–566. 10.1002/glia.22756 25297800

[B49] Lalancette-Hébert M. Gowing G. Simard A. Weng Y. C. Kriz J. (2007). Selective ablation of proliferating microglial cells exacerbates ischemic injury in the brain. J. Neurosci. 27, 2596–2605. 10.1523/JNEUROSCI.5360-06.2007 17344397 PMC6672496

[B50] Lanzillotta A. Porrini V. Bellucci A. Benarese M. Branca C. Parrella E.. (2015). NF-κB in innate neuroprotection and age-related neurodegenerative diseases. Front. Neurol. 6:98. 10.3389/fneur.2015.00098 26042083 PMC4438602

[B51] Lee Y. Lee S.-R. Choi S. S. Yeo H.-G. Chang K.-T. Lee H. J. (2014). Therapeutically targeting neuroinflammation and microglia after acute ischemic stroke. Biomed. Res. Int. 2014:297241. 10.1155/2014/297241 25089266 PMC4095830

[B52] Li L. Lundkvist A. Andersson D. Wilhelmsson U. Nagai N. Pardo A. C.. (2008). Protective role of reactive astrocytes in brain ischemia. J. Cereb. Blood Flow Metab. 28, 468–481. 10.1038/sj.jcbfm.9600546 17726492

[B53] Liu Z. Chopp M. (2016). Astrocytes, therapeutic targets for neuroprotection and neurorestoration in ischemic stroke. Prog. Neurobiol. 144, 103–120. 10.1016/j.pneurobio.2015.09.008 26455456 PMC4826643

[B54] Ma C. Wang X. Xu T. Yu X. Zhang S. Liu S.. (2019). Qingkailing injection ameliorates cerebral ischemia-reperfusion injury and modulates the AMPK/NLRP3 inflammasome signalling pathway. BMC Complement. Altern. Med. 19:320. 10.1186/s12906-019-2703-5 31747940 PMC6868863

[B55] Ma Y. Wang J. Wang Y. Yang G.-Y. (2017). The biphasic function of microglia in ischemic stroke. Prog. Neurobiol. 157, 247–272. 10.1016/j.pneurobio.2016.01.005 26851161

[B56] Mattson M. P. Meffert M. K. (2006). Roles for NF-κB in nerve cell survival, plasticity, and disease. Cell Death Differ. 13, 852–860. 10.1038/sj.cdd.4401837 16397579

[B57] Meffert M. K. Baltimore D. (2005). Physiological functions for brain NF-κB. Trends Neurosci. 28, 37–43. 10.1016/j.tins.2004.11.002 15626495

[B58] Meffert M. K. Chang J. M. Wiltgen B. J. Fanselow M. S. Baltimore D. (2003). NF-κ B functions in synaptic signaling and behavior. Nat. Neurosci. 6, 1072–1078. 10.1038/nn1110 12947408

[B59] Mettang M. Reichel S. N. Lattke M. Palmer A. Abaei A. Rasche V.. (2018). IKK2/NF-κB signaling protects neurons after traumatic brain injury. FASEB J. 32, 1916–1932. 10.1096/fj.201700826R 29187362 PMC5893169

[B60] Mohamadi Y. Mousavi M. Khanbabaei H. Salarinia R. Javankiani S. Hassanzadeh G.. (2018). The role of inflammasome complex in ischemia-reperfusion injury. J. Cell. Biochem. [Epub ahead of print]. 10.1002/jcb.27368 30548879

[B61] Murphy T. H. (2015). Two-photon imaging of neuronal structural plasticity in mice during and after ischemia. Cold Spring Harb. Protoc. 2015, 548–557. 10.1101/pdb.prot087486 26034310

[B62] Nakamura K. Shichita T. (2019). Cellular and molecular mechanisms of sterile inflammation in ischaemic stroke. J. Biochem. 165, 459–464. 10.1093/jb/mvz017 30796426

[B63] Napetschnig J. Wu H. (2013). Molecular basis of NF-κB signaling. Annu. Rev. Biophys. 42, 443–468. 10.1146/annurev-biophys-083012-130338 23495970 PMC3678348

[B64] Nurmi A. Lindsberg P. J. Koistinaho M. Zhang W. Juettler E. Karjalainen-Lindsberg M. L.. (2004). Nuclear factor-κB contributes to infarction after permanent focal ischemia. Stroke 35, 987–991. 10.1161/01.STR.0000120732.45951.26 14988572

[B65] O’Mahony A. Raber J. Montano M. Foehr E. Han V. Lu S. M.. (2006). NF-κB/Rel regulates inhibitory and excitatory neuronal function and synaptic plasticity. Mol. Cell Biol. 26, 7283–7298. 10.1128/MCB.00510-06 16980629 PMC1592877

[B66] Patel S. (2018). Danger-associated molecular patterns (DAMPs): the derivatives and triggers of inflammation. Curr. Allergy Asthma Rep. 18:63. 10.1007/s11882-018-0817-3 30267163

[B67] Pekny M. Wilhelmsson U. Tatlisumak T. Pekna M. (2019). Astrocyte activation and reactive gliosis-A new target in stroke? Neurosci. Lett. 689, 45–55. 10.1016/j.neulet.2018.07.021 30025833

[B68] Rajanbabu V. Galam L. Fukumoto J. Enciso J. Tadikonda P. Lane T. N.. (2015). Genipin suppresses NLRP3 inflammasome activation through uncoupling protein-2. Cell. Immunol. 297, 40–45. 10.1016/j.cellimm.2015.06.002 26123077 PMC4556539

[B69] Ridder D. A. Schwaninger M. (2009). NF-κB signaling in cerebral ischemia. Neuroscience 158, 995–1006. 10.1016/j.neuroscience.2008.07.007 18675321

[B70] Sakai S. Shichita T. (2019). Inflammation and neural repair after ischemic brain injury. Neurochem. Int. 130:104316. 10.1016/j.neuint.2018.10.013 30342960

[B71] Savage C. D. Lopez-Castejon G. Denes A. Brough D. (2012). NLRP3-inflammasome activating DAMPs stimulate an inflammatory response in glia in the absence of priming which contributes to brain inflammation after injury. Front. Immunol. 3:288. 10.3389/fimmu.2012.00288 23024646 PMC3444764

[B72] Schroder K. Tschopp J. (2010). The inflammasomes. Cell 140, 821–832. 10.1016/j.cell.2010.01.040 20303873

[B73] Shekhar S. Cunningham M. W. Pabbidi M. R. Wang S. Booz G. W. Fan F. (2018). Targeting vascular inflammation in ischemic stroke: recent developments on novel immunomodulatory approaches. Eur. J. Pharmacol. 833, 531–544. 10.1016/j.ejphar.2018.06.028 29935175 PMC6090562

[B74] Shen L. H. Li Y. Chopp M. (2010). Astrocytic endogenous glial cell derived neurotrophic factor production is enhanced by bone marrow stromal cell transplantation in the ischemic boundary zone after stroke in adult rats. Glia 58, 1074–1081. 10.1002/glia.20988 20468049 PMC3096459

[B75] Shichita T. Ito M. Yoshimura A. (2014). Post-ischemic inflammation regulates neural damage and protection. Front. Cell. Neurosci. 8:319. 10.3389/fncel.2014.00319 25352781 PMC4196547

[B76] Shih R.-H. Wang C.-Y. Yang C.-M. (2015). NF-κB signaling pathways in neurological inflammation: a mini review. Front. Mol. Neurosci. 8:77. 10.3389/fnmol.2015.00077 26733801 PMC4683208

[B77] Snow W. M. Albensi B. C. (2016). Neuronal gene targets of NF-κB and their dysregulation in Alzheimer’s disease. Front. Mol. Neurosci. 9:118. 10.3389/fnmol.2016.00118 27881951 PMC5101203

[B78] Sofroniew M. V. (2015). Astrocyte barriers to neurotoxic inflammation. Nat. Rev. Neurosci. 16, 249–263. 10.1038/nrn3898 25891508 PMC5253239

[B79] Song L. Pei L. Yao S. Wu Y. Shang Y. (2017). NLRP3 inflammasome in neurological diseases, from functions to therapies. Front. Cell. Neurosci. 11:63. 10.3389/fncel.2017.00063 28337127 PMC5343070

[B80] Strecker J.-K. Schmidt A. Schäbitz W. R. Minnerup J. (2017). Neutrophil granulocytes in cerebral ischemia—evolution from killers to key players. Neurochem. Int. 107, 117–126. 10.1016/j.neuint.2016.11.006 27884770

[B81] Sun S.-C. (2012). The noncanonical NF-κB pathway. Immunol. Rev. 246, 125–140. 10.1111/j.1600-065X.2011.01088.x 22435551 PMC3313452

[B82] Sun S.-C. (2017). The non-canonical NF-κB pathway in immunity and inflammation. Nat. Rev. Immunol. 17, 545–558. 10.1038/nri.2017.52 28580957 PMC5753586

[B83] Sutterwala F. S. Haasken S. Cassel S. L. (2014). Mechanism of NLRP3 inflammasome activation. Ann. N Y Acad. Sci. 1319, 82–95. 10.1111/nyas.12458 24840700 PMC4074217

[B84] Tamura Y. Torigoe T. Kukita K. Saito K. Okuya K. Kutomi G.. (2012). Heat-shock proteins as endogenous ligands building a bridge between innate and adaptive immunity. Immunotherapy 4, 841–852. 10.2217/imt.12.75 22947010

[B85] Voet S. Srinivasan S. Lamkanfi M. van Loo G. (2019). Inflammasomes in neuroinflammatory and neurodegenerative diseases. EMBO Mol. Med. 11:e10248. 10.15252/emmm.201810248 31015277 PMC6554670

[B86] Wang C. Y. Guttridge D. C. Mayo M. W. Baldwin A. S. Jr. (1999). NF-κB induces expression of the Bcl-2 homolog A1/Bfl-1 to preferentially suppress chemotherapy-induced apoptosis. Mol. Cell Biol. 19, 5923–5929. 10.1128/mcb.19.9.5923 10454539 PMC84448

[B87] Wang C. Y. Mayo M. W. Korneluk R. G. Goeddel D. V. Baldwin A. S. Jr. (1998). NF-κB antiapoptosis: induction of TRAF1 and TRAF2 and c-IAP1 and c-IAP2 to suppress caspase-8 activation. Science 281, 1680–1683. 10.1126/science.281.5383.1680 9733516

[B88] Wen H. Miao E. A. Ting J.-P. (2013). Mechanisms of NOD-like receptor-associated inflammasome activation. Immunity 39, 432–441. 10.1016/j.immuni.2013.08.037 24054327 PMC3835203

[B89] West A. P. Shadel G. S. (2017). Mitochondrial DNA in innate immune responses and inflammatory pathology. Nat. Rev. Immunol. 17, 363–375. 10.1038/nri.2017.21 28393922 PMC7289178

[B90] World Health Organization. (2018). Fact Sheets, the Top 10 Causes of Death. Geneva: World Health Organization. Available online at: https://www.who.int/news-room/fact-sheets/detail/the-top-10-causes-of-death.

[B91] Wu M.-Y. Yiang G.-T. Liao W.-T. Tsai A.-P. Cheng Y.-L. Cheng P.-W.. (2018). Current mechanistic concepts in ischemia and reperfusion injury. Cell. Physiol. Biochem. 46, 1650–1667. 10.1159/000489241 29694958

[B92] Xiong X.-Y. Liu L. Yang Q.-W. (2016). Functions and mechanisms of microglia/macrophages in neuroinflammation and neurogenesis after stroke. Prog. Neurobiol. 142, 23–44. 10.1016/j.pneurobio.2016.05.001 27166859

[B93] Yang C. Hawkins K. E. Doré S. Candelario-Jalil E. (2019). Neuroinflammatory mechanisms of blood-brain barrier damage in ischemic stroke. Am. J. Physiol. Cell Physiol. 316, C135–C153. 10.1152/ajpcell.00136.2018 30379577 PMC6397344

[B94] Yang L. Tao L.-Y. Chen X.-P. (2007). Roles of NF-κB in central nervous system damage and repair. Neurosci. Bull. 23, 307–313. 10.1007/s12264-007-0046-6 17952141 PMC5550580

[B96] Zhang S. (2019). Microglial activation after ischaemic stroke. Stroke Vasc. Neurol. 4, 71–74. 10.1136/svn-2018-000196 31338213 PMC6613941

[B95] Zhang H. Ofengeim D. Shi Y. Zhang F. Hwang J. Y. Chen J.. (2016). “Molecular and cellular mechanisms of ischemia-induced neuronal death,” in Stroke: Pathophysiology, Diagnosis, and Management, eds Grotta J. Albers G. Broderick J. Kasner S. Lo E. Sacco R. Wong L. (Philadelphia: Churchill Livingstone Elsevier), 60–79.

[B97] Zhang W. Potrovita I. Tarabin V. Herrmann O. Beer V. Weih F.. (2005). Neuronal activation of NF-κB contributes to cell death in cerebral ischemia. J. Cereb. Blood Flow Metab. 25, 30–40. 10.1038/sj.jcbfm.9600004 15678110

